# Extending standard testing period in honeybees to predict lifespan impacts of pesticides and heavy metals using dynamic energy budget modelling

**DOI:** 10.1038/srep37655

**Published:** 2016-12-20

**Authors:** H. Hesketh, E. Lahive, A. A. Horton, A. G. Robinson, C. Svendsen, A. Rortais, J.- L. Dorne, J. Baas, D. J. Spurgeon, M. S. Heard

**Affiliations:** 1 Centre for Ecology & Hydrology, MacLean Building, Benson Lane, Wallingford, Oxfordshire, OX10 8BB, UK; 2 European Food Safety Authority, 1a, Via Carlo Magno, 1A, 43126 Parma PR, Italy

## Abstract

Concern over reported honeybee (*Apis mellifera* spp.) losses has highlighted chemical exposure as a risk. Current laboratory oral toxicity tests in *A. mellifera* spp. use short-term, maximum 96 hour, exposures which may not necessarily account for chronic and cumulative toxicity. Here, we use extended 240 hour (10 day) exposures to examine seven agrochemicals and trace environmental pollutant toxicities for adult honeybees. Data were used to parameterise a dynamic energy budget model (DEBtox) to further examine potential survival effects up to 30 day and 90 day summer and winter worker lifespans. Honeybees were most sensitive to insecticides (clothianidin > dimethoate ≫ tau-fluvalinate), then trace metals/metalloids (cadmium, arsenic), followed by the fungicide propiconazole and herbicide 2,4-dichlorophenoxyacetic acid (2,4-D). LC_50_s calculated from DEBtox parameters indicated a 27 fold change comparing exposure from 48 to 720 hours (summer worker lifespan) for cadmium, as the most time-dependent chemical as driven by slow toxicokinetics. Clothianidin and dimethoate exhibited more rapid toxicokinetics with 48 to 720 hour LC_50_s changes of <4 fold. As effects from long-term exposure may exceed those measured in short-term tests, future regulatory tests should extend to 96 hours as standard, with extension to 240 hour exposures further improving realism.

Many agricultural and natural ecosystems rely heavily on bees for pollination services. While wild bees are acknowledged to be extremely important pollinators for many plant species e.g. ref. 1, honeybees (*Apis mellifera* spp.) remain the most economically and easily managed pollinator of the main crop monocultures worldwide^2^. Recent widely reported losses of honeybee populations e.g. ref. 3 have been mirrored by declines of wild bees^4^,^5^, with multifactorial effects of stressors such as climate, pathogens, pests (particularly the parasitic mite *Varroa destructor*) and predators, habitat loss and agricultural intensification recognised as influencing bee decline^6^,^7^. In addition, exposure to chemical contaminants including neonicotinoid pesticides and industrial chemicals have been highlighted as posing a significant risk to bees through numerous exposure routes. These include acaracide applications within hives to control parasites and through foraging worker bee exposure to contaminated dust, guttation water, pollen and nectar^4^,^8^,^9^,^10^,^11^.

Ecotoxicological laboratory tests have focused on the honeybee *Apis mellifera* spp. as a surrogate test species for insect pollinators^10^,^12^,^13^, mostly following standardised methods^14^,^15^,^16^,^17^. These typically advise short-term exposure tests (up to 48 h, extended to a maximum of 96 h) to determine acute oral chemical toxicity^18^. However, these short exposure tests do not account for chronic, cumulative effects of exposure to toxicants, despite the fact that time-dependent toxicity of pesticides is described in honeybees^19^,^20^. Therefore, chronic toxicity tests are necessary to provide dose-response data of cumulative, long-term exposure effects, to include field realistic low and sub-lethal chemical doses. Such data at the individual level, will be essential to understand the impact of chemicals on bee mortality over extended exposure to chemicals and to better inform risk assessment for colonies and populations.

Recognising this need, the European Food Safety Authority (EFSA) published a Scientific Opinion^13^ that considered key issues for hazard risk assessment for bees. This identified future research should be focussed on both acute and chronic toxicological studies for different classes of chemicals (and their metabolites) for both lethal and sub-lethal effects^13^. EFSA also recommended a requirement for standardised laboratory tests to determine these effects on lethal endpoints to chemicals and contaminants over longer time-periods than the existing OECD guidelines. To address both this issue and the dearth of data for many chemicals in respect of chronic exposures, we developed a combined acute and chronic oral toxicity test in adult honeybees that extends the standard OECD protocol from 96 h acute toxicity tests to a 240 h (10 day) chronic exposure. We used this test to examine toxicity to honeybees of different chemicals that reflect both current concerns about agrochemicals and trace pollutants in the environment.

Based on the extended test data, for the first time in bees we used a Dynamic Energy Budget toxicity model (DEBtox) to improve understanding of chemical effects on individuals^21^,^22^,^23^. Such process-based approaches can provide better insights into the mechanisms and long-term effects of stressor exposure compared to standard methods e.g. probit analyses to determine LCx or ECx^24^,^25^ (the lethal concentration or exposure concentration killing or affecting a certain proportion “x” of individuals at a defined endpoint). In contrast to standard probit analysis, which disregards data that does not refer to a specific ECx or LCx endpoint, DEBtox models use all available information from across exposure experiments i.e. survival data for all time points and all concentrations, with chemical concentration as a time invariant exposure metric^26^,^27^. The DEBtox model is based on DEB (Dynamic Energy Budget) theory which systematically incorporates the exposure time to chemicals with the biology of the organism including life-cycle information on feeding, maintenance, growth, development and reproduction. The model describes both toxicokinetics (quantification of metabolic and elimination processes) and toxicodynamics (toxic dose responses) which can provide understanding of time-related effects^28^,^29^,^30^. Whilst allowing calculation of standard LC_50_ values similar to probit analysis, DEB models provide significantly more powerful abilities to predict chemical effects and describe toxicity dynamics. When linked with population dynamic processes, DEBtox can be used to model impacts on the life-span expectancy of individual bees. Since toxic effects are interpreted as time-dependent parameters, they can be used to predict either short-term or long-term effects on key traits linked to population parameters^23^. DEB models can therefore be extremely useful and powerful tools to extrapolate toxic effects for single compounds measured at the individual level, to meaningful consequences at the population level^24^,^31^.

Here, we examine the differential effects of seven chemicals with different modes of metabolic action on honeybees. These chemicals were selected to explore how variation in toxicokinetic and toxicodynamic processes alter accumulative effects on bee mortality for exposure periods longer than the current OECD recommended test duration. We compared results from data analysis using the DEBtox model to standard toxicity test results generated survival data, collected daily for up to 240 h exposure in order to; 1) determine whether standard analysis recommended for acute 96 h exposure tests is experimentally adequate to describe longer term impacts of chemicals to 240 h; 2) to better understand how the toxicokinetics and toxicodynamics differ between chemicals with respect to accumulation/elimination effects within bees using the DEBtox model and; 3) extrapolate the toxic effects for single chemicals using the DEBtox model to predict life span toxicity effects using survival data in 240 h tests for adult worker honeybees that may be expected to represent the average lifetime of worker bees in summer and winter.

## Results

We conducted acute oral toxicity testing on *Apis mellifera* spp. for seven different chemicals, including insecticides, pesticides and industrial chemicals (Table 1). Groups of ten adult worker bees from four replicate colonies were allowed to feed *ad libitum* up to 240 h on sterile sucrose solution spiked with a range of concentrations for each tested chemical and compared to untreated sucrose solution (±1% acetone solvent). Data were analysed using probit regression and to parameterise DEBtox models, from which physiological, toxicokinetic and toxicodynamic trait parameters were derived. Estimates of effect concentrations (LC_50_, LC_5_) were derived from the short-term toxicity data and extended to model lifespan exposure effects for adult worker bees.

### Acute and chronic effects of chemicals to bees over 10 day continual exposures

**Figure 1:**
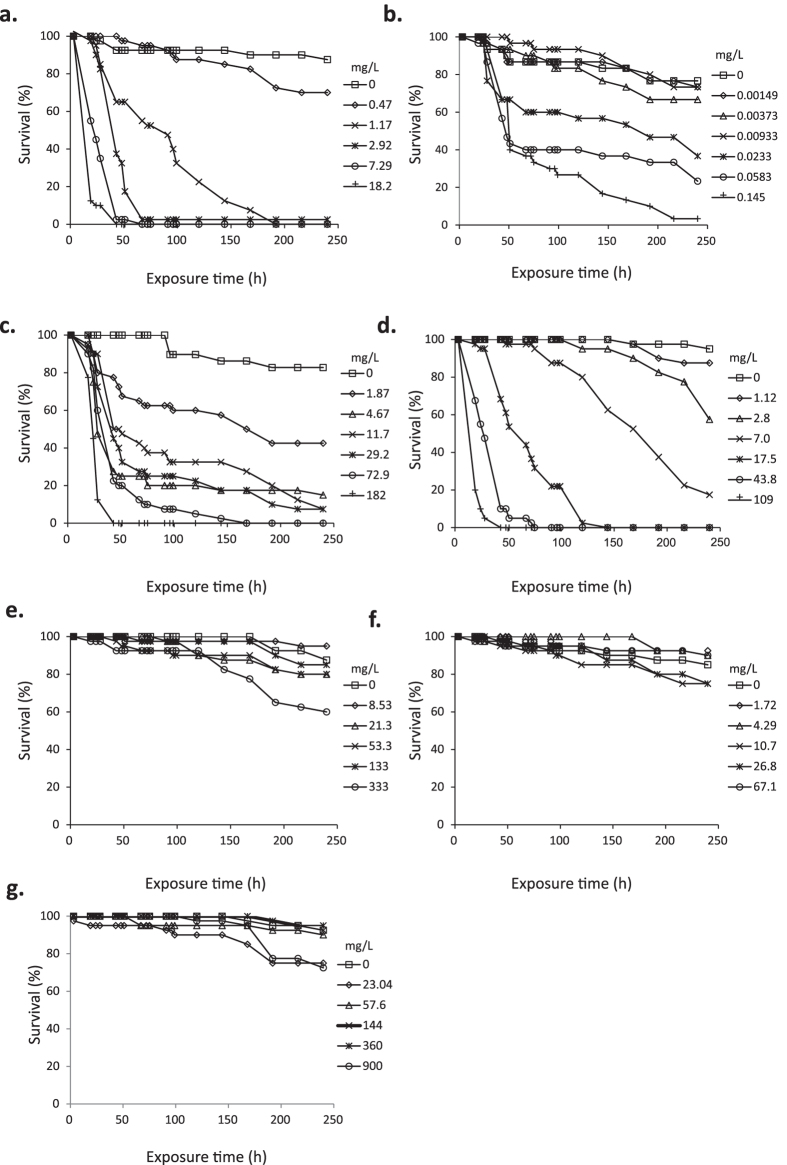
Survival patterns in time given as percent bees surviving (n = 40 bees tested per chemical concentration with each replicate (n = 4) comprising a group of 10 bees from each of four different colonies) of *Apis mellifera* spp. exposed to a series of concentrations of (**A**) dimethoate; (**B**) clothianidin; (**C**) cadmium; (**D**) arsenic; (**E**) propiconazole; (**F**) tau-fluvalinate; (**G**) 2,4-D by a 240 h continuous oral exposure.

Patterns of effects on bee survival over time differed between the tested chemicals (Fig. 1a–g) with significant concentration-mortality effects for dimethoate (Fig. 1a), clothianidin (Fig. 1b), cadmium (Fig. 1c) and arsenic (Fig. 1d). There was low control mortality across tests; average control mortality in sucrose only and acetone control groups for all chemicals tested was 5.02% after 96 h following start of exposure (less than 10%, as recommended for acute, oral toxicity tests in bees^14^), increasing to 7.34% at 7 d and 12.74% at 240 h (less than 15%, as a suggested recommendation for chronic 10 day oral toxicity testing in bees^17^). In a single test with clothianidin, control mortality at 96 h equalled 13.33% and then exceeded the recommended 15% at 240 h, but this is accounted for within the DEB tox analysis. The calculated probit and DEBtox LC_50_ values at 48, 96 and 240 h for these four chemicals were directly comparable to conventional probit calculated values for the same time-points as indicated by a strong, significant correlation between LC_50_ values across the three time points calculated by the two methods (regression analysis of Log_10_ transformed DEBtox LC_50_ against Log_10_ transformed probit LC_50_ values (mg/L); F_1,9_ = 130.5; p < 0.001; r_2_ = 0.94; regression equation log_10_DEBtox = -0.117 + 1.20 Log_10_probit; intercept not significantly different from zero, t = -1.165, p = 0.274). For completeness and comparability with previous studies, LC_50_ values calculated using the two methods of analysis are reported in Table 2.

**Figure 2:**
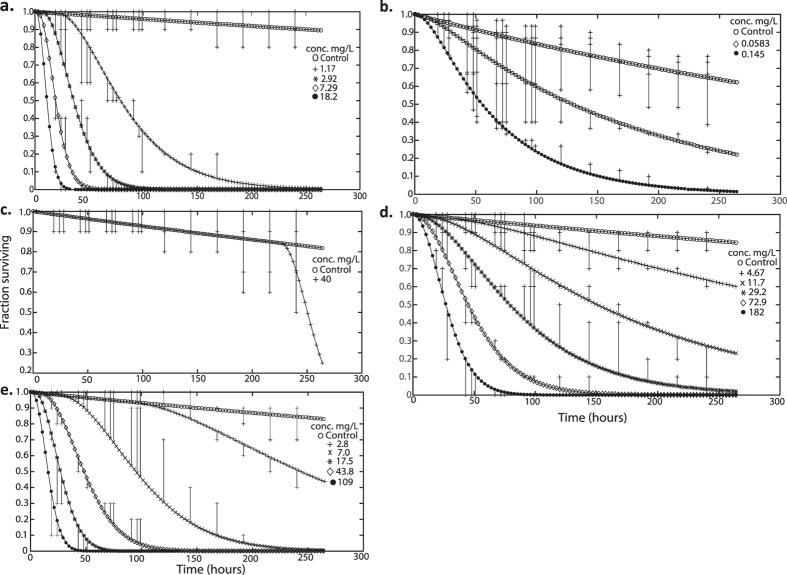
Measured (+) time course of mortality and fitted (−) DEBtox model estimates of survival response over time for *A. mellifera* spp. exposed to a series of concentrations of (**A**) dimethoate; (**B**) clothianidin; (**C**) propiconazole; (**D**) cadmium; (**E**) arsenic over a 240 h continuous oral exposure. Vertical lines indicate the difference between the observed data relative to the model output.

**Figure 3:**
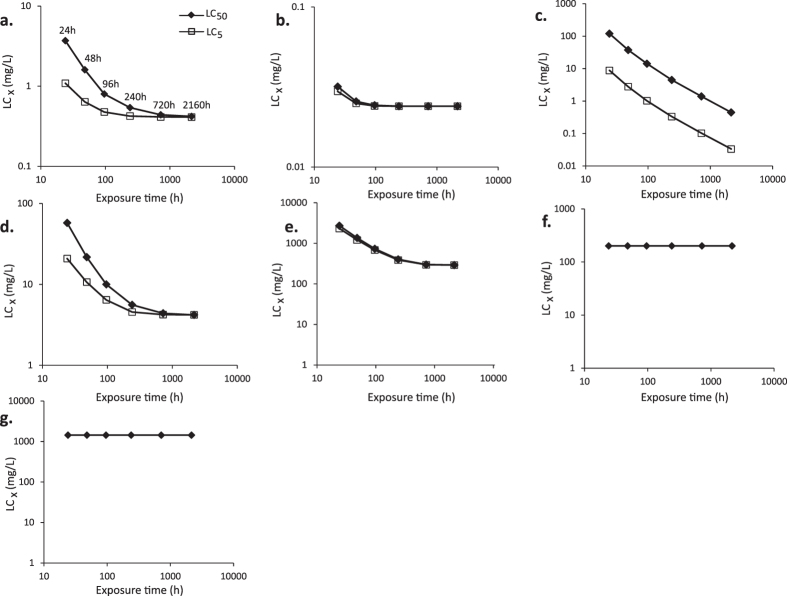
Relationship between LC_50_ (closed diamonds, solid line) and LC_5_ (open squares, dashed line) values estimated from DEBtox model parameters for *Apis mellifera* spp. exposed to a series of concentrations of (**A**) dimethoate; (**B**) clothianidin; (**C**) cadmium; (**D**) arsenic; (**E**) propiconazole; (**F**) tau-fluvalinate; and (**G**) 2,4-D and exposure time estimated for time periods (24, 48, 96, 240 h) relating to the exposure and predicted for extended exposure time relevant to the life-span of a worker bee during normal summer season (720 h) and when overwintering (2160 h).

There were significant concentration-mortality effects at all time points for the reference chemical dimethoate (Fig. 1a; Fig. 2; Supplementary Table S1). Probit calculated LC_50_ values ranged from 2.42–0.62 mg/L at 48 h to 240 h respectively with comparable DEB calculated LC_50_ values of 1.55 mg/L at 48 h and 0.54 mg/L at 240 h (Table 2). The 48 h probit LC_50_ of 2.42 mg/L (95% CI 1.96–2.89; Table 2) equates to an estimated LD_50_ of 3.39 × 10^−4^ (95% CI 2.74 × 10^−4^– 4.04 × 10^−4^) mg/bee based on an average measured consumption rate of 0.07 mg/bee/day (Supplementary Table S2). This approximates to the upper limit of the range of the oral LD_50_ values at 24 h of 1.0 × 10^−4^–3.5 × 10^−4^ mg/bee for dimethoate as the standard positive control in honeybee toxicity tests^14^. The comparatively high killing rate for dimethoate underpins strong time dependent effects on survival (Fig. 3a); if the killing rate is infinitely high, death is immediate once the no effect concentration (NEC) (a toxicological threshold below which no effect occurs for any exposure time) is exceeded. The effects of time on DEB LC_50_ and LC_5_ values over experimental (24, 48, 96, 240 h) and maximum summer and winter worker life-span time-points of 720 h (30 days) and 2160 h (90 days) showed that the LC_50_ and LC_5_ converge closer to the NEC with extended exposure time (Fig. 3a). Dimethoate LC_50_ and LC_5_ values approached the NEC of 0.41 mg/L within the test duration, with the DEB LC_50_ being within a factor of two and one of the NEC after the 96 h and 240 h, respectively, and predicted to approximate to the NEC for 720 h and 2160 h exposures (Fig. 3a). This suggests that sensitivity for dimethoate indicated by deriving an LC_50_ from a short-term 96 hour exposure would be within an order of magnitude of that likely to result from a full lifetime dimethoate exposure for a summer or winter worker bee.

For clothianidin, there were two sets of parameter values with an equally good fit to observed data. One parameter set had a low NEC of 0.0054 mg/L (with low blank killing rate) and the second had a NEC of 0.024 mg/L (with higher blank mortality). These NEC values were compared to the DEB fits for individual colonies and also for two, independent data-sets with clothianidin exposure data that were conducted in a later study. Individual NEC values for colonies in the current study were; 0.019, 0.027 and 0.045 mg/L whilst the NEC values for the two independent data-sets were 0.038 and 0.053 mg/L. We based further analysis on the DEB data-set with a NEC of 0.024 mg/L as the most biologically plausible, being within the range of independent colony NEC values and closer to the NEC values for the independent data. A low NEC of 0.024 mg/L reflected honeybee sensitivity to this chemical. Elimination rate values predicted that 95% of equilibrium body burden of clothianidin will be reached after approximately 41 h; the high killing rate based on this internal concentration defines a rapid progression of toxicity with time. There was significant mortality in bees exposed to concentrations of 0.0037 mg/L and above, compared to controls (Fig. 1b; Fig. 2; Supplementary Table S1). Calculated probit LC_50_ values decreased with time from 0.158 mg/L after 48 h, to 0.079 mg/L at 96 h and finally 0.028 mg/L after 240 h (Table 2). Using DEBtox parameters to estimate LC_5_ and LC_50_ values over the exposure highlights how these metrics rapidly approach the NEC (Fig. 3b). For example, the 96 h and 240 h DEB LC_50_, along with the predicted 720 h and 2160 h LC_50_ values are equivalent to the NEC. Hence short-term test results of 96 h to derive an LC_50_ would provide an indication of sensitivity within an order of magnitude of that occurring for workers as a result of lifetime exposure (Fig. 3b).

Cadmium produced significant dose-mortality responses at all time points and there were some differences between colonies in the first 48 h of exposure, but not at 96 h or 240 h (Supplementary Table S1). The rapid initial mortality in bees at the highest concentration was followed progressively over time by high mortality in even the lowest concentration tested (Figs 1c and 2). The probit calculated LC_50_ value for cadmium of 18.36 mg/L at 48 h therefore reduced rapidly to 3.70 mg/L at 96 h. This high toxicity was reflected in the DEBtox NEC for cadmium which was equivalent to zero (1 × 10^−7^ mg/L), indicating that there is no level of exposure that would not, over a sufficient exposure time, result in mortality above the background rate (Fig. 3c). The elimination rate of 0.037 h^−1^ indicates bees will take 80 h to reach 95% of internal equilibrium. Even though accumulation progresses to equilibrium well within the exposure period, mortality progresses relatively slowly due to the low killing rate. As an example, the DEB LC_50_ reduces from 37.7 mg/L to 4.5 mg/L when exposure time increases from 48 h to 240 h. Further reductions of LC_50_ values to 1.4 mg/L are predicted for 720 h exposure and to 0.45 mg/L for 2160 h exposure (Fig. 3c). This corresponds to a predicted >25 fold reduction in LC_50_ when the exposure period is extended from a 48 h laboratory test duration to a full adult worker life-span. Even with the extent of reduction with time, the LC_50_ remains above the NEC. For this chemical alone, the LC_5_ DEBtox parameters were also estimated for 48 h, 96 h, 240 h, and 720 h exposure times as 2.79 mg/L, 1.02 mg/L, 0.33 mg/L and 0.1 mg/L respectively.

For bees exposed to arsenic, all bees were dead in the top two exposure concentrations after 96 h (Figs 1d and 2) and there was a significant dose-mortality effect at all time points (Supplementary Table S1). Concentration and time dependent effects on mortality were reflected by probit LC_50_ values of 25.7 mg/L after 48 h and 4.03 mg/L after 240 h. The NEC estimated for arsenic was 4.2 mg/L and DEB analysis identified differences between colonies. These covered a factor of 3, with the lowest colony NEC being 1.74 mg/L and the highest 5.6 mg/L. As for clothianidin, this difference may be explained by variations in individual colony sensitivity. The elimination rate predicts that it takes 200 h time to reach 95% of internal equilibrium concentration. The killing rate for arsenic is, however, relatively low, being higher only than that for cadmium. Hence although internal equilibrium is reached during the exposure period, toxicity progresses comparatively slowly as illustrated by time dependent LC_5_ and LC_50_ values (Fig. 3d). Thus, the LC_50_ at 96 h remains twice that of the NEC. When extended to 720 h exposure, the DEBtox predicted LC_5_ and LC_50_ values approach the NEC (Fig. 3d).

Propiconazole did not produce a clear concentration dependent effect at any concentration for time-points up to 144 hr but exposure to the highest test concentration (333 mg/L) significantly increased mortality up to 40% at 240 h (Supplementary Table S1), suggesting an LC_50_ close to the top tested concentration. DEB analysis showed an effect on survival in three of four colonies only at the highest tested concentration. The NEC for propiconazole was 292 mg/L which is close to the highest tested concentration and exceeds those for dimethoate and clothianidin by 3 and 4 orders of magnitude respectively. The elimination rate estimated for propiconazole indicates that bees will take 166 h to reach 95% of internal equilibrium. A relatively low killing rate results in a slow progression of toxicity in time when the NEC is exceeded. After 240 h exposure, there was less than 50% mortality at the top exposure dose of 333 mg/L. Using DEBtox to predict the LC_50_ values for 720 h and 2160 h exposure times indicates values that relate closely to the NEC (Fig. 3e). There was no clear effect of concentration for the remaining two chemicals; neither tau-fluvalinate (Figs 1f and 3f) or 2,4-D (Figs 1g and 3g) affected survival at 48 h, 96 h or 240 h (Supplementary Table S1).

The fits identify large differences in NEC values relating to the potency of the three insecticides (i.e. dimethoate, clothianidin and tau-fluvalinate (assuming NEC above the highest test concentration for tau-fluvalinate), as well as differences in toxicokinetic and toxicodynamic traits that influence the pattern of toxicity in time. The NEC for dimethoate of 0.41 mg/L is an order of magnitude higher than that for clothianidin 0.024 mg/L indicating an intrinsic lower potency for the organophosphate. A slightly slower elimination rate is derived for dimethoate than clothianidin (0.04 *vs* 0.073 h^−1^, respectively). Based on this value, internal dimethoate concentrations take approximately 75 h to reach 95% of equilibrium.

A wide-range of changes in the pattern of sensitivity in relation to exposure time was indicated by comparison of calculated LC_50_ values for the different tested chemicals. Comparing between values for different time points showed change by a factor of between 1.07 and 8.34 for a comparison of the 48 h: 240 h LC_50_ values across the 7 chemicals, between 1.07 and 27.11 for the 48 h: 720 h comparisons and between 1.07 and 83.73 for the 48 h: 2160 h comparison (Table 2). The highest short: long-term LC_50_ ratios were for cadmium indicating highly time dependent toxicity for this metal. Time dependence toxicity for dimethoate, clothianidin, propiconizole and arsenic indicated changes in toxicity and effects on survival of approximately 3–5 fold.

## Discussion

This is the first report to our knowledge that links toxicokinetic and toxicodynamic processes of multiple chemicals in honeybees using Dynamic Energy Budget toxicity (DEBtox) models. Previous studies have considered simple toxicological models to estimate long-term effects of pesticides in multiple species and results suggest that in all cases, lethal effects accumulate in insects^20^. Current laboratory acute oral toxicity tests in *A. mellifera* spp. use short-term exposures, to a maximum of 96 h, which do not account for chronic cumulative toxicity. For most of the chemicals we tested, our results indicate that acute tests of at least up to 96 h duration are suitable to estimate the environmental exposure concentration that will have no significant effect on mortality with indefinite exposure time i.e. the NEC (no effect concentration). However, the effects of chemicals that accumulate over time or exhibit delayed toxicity are unlikely to be identified under the present, regulatory guidelines for acute, short exposure studies of up to 96 h. Indeed, this study demonstrates that for the trace metals cadmium and arsenic, additional data may be required through extended testing periods beyond 96 h, as delayed lethal effects over time are related to the specific accumulation and elimination of these chemicals in honeybees.

Published toxicity data on clothianidin, tau-fluvalinate, dimethoate, arsenic and cadmium for *A. mellifera* spp. showed variation among studies and no results were found for 2,4-D and propiconazole. Therefore, these results are the first to present chronic oral toxicity data for 2,4-D and propiconizole and the most recent for arsenic. There are numerous additional advantages in using DEBtox models for the analysis of this type of toxicity data, even where only survival is recorded^32^,^33^,^34^,^35^,^36^,^37^,^38^. In the DEBtox model structure, there is an intimate relationship between survival and toxicokinetics; uptake of the chemical to a level above the NEC is required for effects on survival above background mortality to occur. The NEC provides a threshold for toxicity independent of exposure time, so is an ideal parameter through which to compare chemical potency as it avoids issues related to the time dependency of classical parameters like LC_x_/EC_x_ values^24^,^39^,^40^; outputs from the DEBtox model give mechanistic parameters which describe toxic potency of a compound once the NEC is exceeded. These parameters provide information on relevant physiological processes including metabolism and chemical related damage as well as the ability to calculate any LC_x_ for any point in time, including exposure durations beyond the test time-frame.

The LD_50_ value we identified for clothianidin at 96 h was 2.21 × 10^−5^ mg/bee (based on assumed 0.07 ml/bee/day consumption rate) and is comparable with previously published results for *A. mellifera* spp. This includes a 24 h LD_50_ of 2.18 × 10^−5^ mg/bee identified by Iwasa *et al*.^28^ and the 24–72 h LD_50_ 1 × 10^−6^–7 × 10^−6^ mg/bee reported in Laurino *et al*.^30^. Differences between studies may be because Laurino *et al*.^30^ used a commercial clothianidin formulation, whilst the active ingredient alone was used in this study^29^. It is also possible that colony specific differences in detoxification capacity influence these observed differences as well as other within-colony or inter-(sub) species variations in sensitivity^12^,^41^. Indeed, a high sensitivity of *A. mellifera* spp. to clothianidin was identified as a low NEC (0.024 mg/L) was calculated. The DEBtox derived elimination rate provides key evidence relating to the overall internal fate of the chemical and for clothianidin indicates it is detoxified quickly, through relatively rapid metabolism. This is similar to previous studies in which the assimilation of another neonicotinoid, imidacloprid, has suggested a relatively high rate of detoxification in *A. mellifera* spp. when compared to bumblebees^42^. Our results are strikingly consistent with those recently reported for exposure of winter bees to clothianidin in 10 day, chronic exposure oral toxicity tests, despite the fact that the bees used in the current study are summer workers^43^. The LD_50_ value reported by Alkassab & Kirchner at 96 h of 1.51 × 10^−5^ mg/bee is similar to that from our test (based on the assumed consumption rate above) of 2.21 × 10^−5^ mg/bee and similarly, the probit calculated LD_50_ at 240 h in our study was 1.96 × 10^−5^ mg/bee whilst Alkassab & Kirchner reported a 10 d LD_50_ of 9.5 × 10^−6^ mg/bee. Given the similarity of these results, the DEB LD_50_ of 0.024 mg/L for 2160 h could approximate well for exposure across the lifespan of a winter bee, demonstrating the chronic exposure levels that may have effect on over-wintering colonies, although experimental data would be required to validate this.

A NEC was not calculated for tau-fluvalinate but can be expected to be orders of magnitude higher than those for the other insecticides as there was hardly any mortality in these treatments. This low sensitivity of *A. mellifera* spp. to tau-fluvalinate can be linked to rapid detoxification^44^ through the cytochrome P450 monooxygenases (P450s) enzyme pathway. Similarly, the highest rate of elimination for dimethoate is through metabolism driven by enzymes including cytochrome p450s^45^. This effective detoxification allows wide use of tau-fluvalinate as a miticide that is considered safe to bees. That said, in this study we found this chemical to be less toxic in comparison to studies by Johnson *et al*.^46^. This is likely due to exposure route and dosing methods; we administered the dose orally whilst Johnson *et al*.^46^ used topical applications and our exposures were continuous compared to spiked exposure tests in other studies. Consequently, a lethal dose was not achieved in this study as our test exposure concentrations were limited by solubility of tau-fluvalinate.

The NEC for propiconazole was higher than clothianidin or dimethoate by orders of magnitude and we found low toxicity at a concentration near to maximum water solubility. Similarly, we found no toxicity for 2,4-D at the upper limit to water solubility which is the first concentration related toxicity information that we are aware of for this herbicide, other than herbicide registration documents which report low toxicity for honeybees^47^ with no effect up to 0.01 mg/bee (note that this approximates to 70% of the dose received by feeding bees at our top tested concentration after 48 h exposure). Previous studies of contact and oral toxicity of propiconazole formulations indicate 24 h and 72 h LD_50_ values of 0.0617 mg/bee and 0.0485 mg/bee for *A. mellifera* spp. and 0.0678 mg/bee and 0.0224 mg/bee for *Osmia lignaria*^48^. The fact we found no dose-mortality effect of propiconazole compared to these reported values may be due to use of formulations that cause different adsorption and transport compared to the active ingredient alone used in this study. Indeed, there may even be direct toxicity associated with additives in formulations; for example, an “inert” solvent N-methyl-2-pyrrolidone was found to be highly toxic to honeybee larvae^29^. Whilst we did not detect direct toxicity, the presence of such compounds as part of an environmental mixture may potentially have additive or interactive toxicological effects under field conditions. Currently, mixture toxicity studies with environmentally relevant exposures are lacking for honeybees but those that have investigated binary mixtures of miticides and fungicides suggest synergistic or additive toxicity to larvae and adult bees^13^,^29^. In the same way, whilst it is unlikely that there would be effects of 2,4-D from nectar or drinking water exposure as we detected no toxicity at maximum water solubility, there is still potential for this herbicide to interact with other chemicals in mixtures. With potentially significant impacts on colony health, there is a pressing need to include mixture studies in future risk assessment and toxicity testing.

The two trace elements tested showed differences in their potential elimination. The time course of cadmium effects on survival were consistent with relatively slow elimination, leading to accumulation over an extended time period in a manner synonymous to what is known in other species including humans^49^,^50^,^51^. There was very high sensitivity of bees to cadmium and the NEC for this metal was effectively zero, which indicates there is no safe limit of exposure for bees to this chemical. Due to the relatively low toxicokinetic and toxicodynamics of cadmium the full effect of toxicity may not be realised within a worker bee life-span. For cadmium, the DEBtox predicted LC_5_ of 1 × 10^−4^ mg/L for maximum life-span (720 h and 2160 h for summer and winter respectively) could be taken as an alternative low threshold value for effects. Elimination for arsenic is even slower than for cadmium, but the relatively low toxicodynamic level indicated by the killing rate means that LC_5_ and LC_50_ values over a time course of exposure approach the NEC. Differences in elimination and killing rates for cadmium and arsenic, suggest different handling for detoxification and possibly varying effects pathways. For both, this may include metallothionein^52^ whilst for arsenic, mechanisms such as methylation may be sufficient to account for increased accumulation over time^53^. Similarly, both trace elements may induce effects through reactive oxygen species production, but may also involve other underlying mechanisms.

Toxicity for the two trace metals in *A. mellifera* spp. is largely consistent with the minimal amount of available toxicity data for these chemicals; Cronn^54^ reported 48 h LD_50_ values in *A. mellifera* spp. ranging from 0.00234–0.00351 mg/bee for cadmium sulphate and 96 h LD_50_ values ranging from 0.00144–0.0028 mg/bee for cadmium chloride. These results for short-term exposure are consistent with the toxicity values determined in the current study based on assumed consumption rates (although longer exposure of 240 h resulted in values up to an order of magnitude lower than those found previously). The only published LD_50_ we found for arsenic was reported in a 70 year old study^55^ of 6 × 10^−4^ mg/bee for exposure to insecticidal arsenate powders. This value is similar to the 96 h LD_50_ estimated from our exposure of 3.79 × 10^−3^ mg/bee (see Supplementary Table S2). Conservatively, for protection from longer term exposure effects a factor of 25 would be needed to account for the temporal changes in effects that would result during long-term exposures for chemicals that have known slow elimination kinetics such as cadmium. Even for chemicals without such slow kinetics a factor of 5–10 may be appropriate, as 48 h and 720 h LC_50_ values rarely fall within a factor of 3. However, even extension to 96 h means that 4 of 5 chemicals would give an LC_50_ within a factor of 5 of the predicted 720 h LC_50_ value, namely dimethoate, clothianidin, propiconazole and arsenic with the exception being cadmium.

Potentially, honeybees may be exposed to pesticides and environmental chemicals over their full life-time. We have demonstrated that sensitivity resulting from long-term exposure indicates a greater hazard to bees than would be assumed from results from current short-term (48 h, 96 h) toxicity tests in *A. mellifera* spp. Extended duration tests to 240 h, coupled with the use of simple process based modelling approaches such as DEBtox models will significantly improve hazard assessment. Recently, a draft guideline has been published for honeybee chronic oral toxicity feeding tests in the laboratory up to 240 h exposures^17^ and our results suggest adoption of this will significantly improve the predictive power of toxicity tests. As a minimum, our results suggest that future regulatory tests extend to 96 h as standard, rather than 48 h. Importantly, hazard assessment should also include understanding of the toxicokinetics and toxicodynamics for different chemicals as this provides a basis for developing population dynamics-based models to predict acute and chronic effects in individuals as a key input for models for colony level effects. Understanding the time-dependency of effects clearly shows that the consequences of long-term exposure can exceed those measured in short-term tests by an order of magnitude or more, with those chemicals with slow toxicokinetics showing the greatest discrepancy between short- and long-term hazard. In this respect, further data are needed to better inform risk assessment and understand the consequences of the effects at the individual level we have described on population levels of honeybees.

## Methods

### Apiary setup

Colonies of *A. mellifera* spp. were established in 2014 and maintained in National Hives at the Centre for Ecology & Hydrology (Wallingford, Oxfordshire, UK). Regular colony inspections were carried out weekly (April-September 2014, 2015), including prevalence of levels of *Varroa destructor* (number of mites) and bee infectious agents, to ensure colonies were queen right, with healthy brood (larvae) and adult bees. Only those hives that contained no visible evidence of pests and/or infectious agents were included in subsequent toxicity tests.

### Chemicals

Dimethoate, clothianidin, propiconazole, tau-fluvalinate, cadmium chloride (cadmium), sodium arsenate dibasic heptahydrate (arsenic) and 2,4-dichlorophenoxyacetic acid (2,4-D) were obtained from Sigma-Aldrich^®^ Ltd. as analytical grade chemicals and pesticide standards (PESTANAL^®^). Acetone solvent used was HPLC-grade and sucrose for feeding solutions was also obtained from Sigma-Aldrich^®^ Ltd. with ≥99.5% purity. The chemical concentrations tested in mg/L (i.e. μg/ml or ppm) were: dimethoate; 0.47, 1.17, 2.92, 7.29, 18.23; clothianidin: 0.00149, 0.00373, 0.00933, 0.0233, 0.0583, 0.145; tau-fluvalinate: 1.72, 4.29, 10.73, 26.83, 67.08; 2,4-D: 23.04, 57.60, 144, 360, 900; propiconazole: 8.53, 21.33, 53.33, 133, 333; cadmium: 1.87, 4.67, 11.67, 29.17, 72.92, 182; and arsenic: 1.12, 2.80, 7.00, 17.50, 43.75, 109. Stock solutions were initially prepared in water or acetone solvent (depending on chemical solubility) and diluted to give the concentrations detailed in feeding solutions of 50% (w/v) aqueous sucrose solution. Sucrose solutions were made up in autoclaved, ultrapure water using molecular grade ≥99.5% GC quality sucrose from Sigma-Aldrich^®^ Ltd. Where acetone was used as a solvent, the concentration was 1% acetone in the feeding solutions.

### Bioassay procedure

Adult worker honeybees were exposed orally to all chemicals. Bioassays followed standard protocols^14^,^15^, with modifications to extend exposure time from 96 h to 240 h and increase temporal monitoring of survival. Even-aged, adult worker honeybees were obtained from one or two frames containing young brood from each of four replicate colonies that were queen right, with no indication of mite or pathogen presence. Cohorts were chilled at −20 °C for a maximum of 45 s to gently anaesthetise bees, prior to loading into test cages. Bees were assayed in cages made from clear plastic pots with a ventilated lid into which a 50 ml slip luer tip syringe (free from latex and silicone oil) containing chemicals was inserted, with the tip of the syringe cut off to allow bees to feed readily. A total of 10 bees were tested in each replicate, for each tested concentration for each of the four colonies, giving a total of 40 bees per chemical concentration exposure. Negative controls were either pure 50% (w/v) sucrose solution or with 50% sucrose solution with 1% acetone as in the test chemical group.

During the experiment, bees were maintained in a controlled environment room at 25 ± 1 °C; 60% relative humidity (as recommended for standardised tests^14^) under constant dark for 240 h and exposed continuously to test chemicals or control solutions. Mortality was recorded three times daily during the 0 h to 96 h exposure period and then once daily until the test ended at 240 h. Syringes were weighed at the start of the experiment on initial exposure to chemicals and then at 48 h, 96 h and 240 h post-exposure, to allow estimation of consumption rate to be calculated.

### Data analysis

Mortality data were analysed using the probit analysis function in Minitab 16 v. 1.0. The LC_50_ values (±95% confidence intervals) i.e. the concentration of chemical required to kill 50% of test bees were calculated for 48 h, 96 h and 240 h exposure time-points to provide an assessment of sensitivity and a comparable toxicity metric to relate to published data. Mortality differences between the proportional mortality in treated and control bees at each time point was compared using the Generalised Linear Model (GLM) function in Minitab 16 v. 1.0. As exposed bees fed continuously, chemical concentration in the sucrose solution was the time-invariant exposure parameter used to compare sensitivity of bees to different chemicals across time points. Chemical intake (i.e. exposure “dose”) was estimated as the mean intake rate per bee, calculated from the consumption per unit time (measured by change in syringe weight between time-points) and survival at each time point. The actual dose received by an individual bee will increase over time and therefore estimated LD_50_ values will also increase, while conversely the LC_50_ can be expected to (at least initially) fall. Dose can also be adjusted by individual body weight to give dose/mg bee tissue to account for any effects of body size on sensitivity. In the current study, results are discussed in relation to LC_50_ values but estimated effect concentrations in relation to body weight are given in Supplementary Table S2 for reference.

DEBtox modelling allowed the concentration and time dependent effects on survival to be described in relation to DEB parameter estimates (Fig. 2a–e; Table 3). Blank hazard rates (i.e. background control mortality) were low (0.0005–0.003 h^−1^) across all tests which was consistent with observed low control mortality rates. For dimethoate, clothianidin, propiconazole, cadmium and arsenic, the DEBtox model was fitted separately for each experimental cohort (i.e across all colonies) and for replicate colonies (Fig. 2; Table 1) which resulted in reliable and robust model fits. Models were not fitted for tau-fluvalinate or 2,4-D, although parameter estimates were possible for some colonies based on partial effects in the highest exposure concentrations. There were inconsistencies in the data for one colony (#3) in the clothianidin data set, characterised by high mortality at two intermediate exposure concentrations. Inclusion of this data lead to a lower NEC estimate than that presented, but was derived from a weaker fit, hence the reported NEC was calculated excluding this colony to ensure robustness of parameter estimates.

The NEC (no effect concentration) within DEBtox provides a threshold for toxicity not dependent on exposure time so is an ideal parameter through which to compare chemical potency as it avoids issues related to the time dependency of classical parameters like LC_x_/EC_x_ values^24^,^39^,^40^. The outputs from DEBtox model provide time-independent, mechanistic parameters which describe toxic potency of the compound once the NEC is exceeded. Knowing these parameters provides information on relevant physiological processes including metabolism and chemical related damage as well as the ability to calculate any LC_x_ for any point in time, including exposure durations beyond the test time-frame. The model used was originally developed by Kooijman and Bedaux^56^ and refined by Jager *et al*.^27^. It takes the form of a scaled one-compartment model to describe uptake and elimination and a hazard model to describe effects on survival. Within the model framework, four time-independent parameters describe the overall survival pattern in time namely 1) the blank killing rate: a measure of the rate of the background mortality in a population not subject to exposure (h^−1^); 2) the no effect concentration (NEC): a time-independent toxicological threshold, expressed as an environmental concentration (mg/L test solution), below which no effects occur even over infinite exposure time; 3) the elimination rate (*k*_*e*_): a rate parameter determining when the equilibrium between internal and external concentration is reached in time (h^−1^) and 4) the killing rate (*k*_*k*_): the toxic potency of the compound (once the NEC is exceeded) expressed in relation to the environmental concentration and time (conc^−1^ h^−1^).

In the model, the NEC is particularly important as this parameter represents the threshold-concentration causing increased hazard (mortality). The NEC denotes the maximum concentration to have no effect on the measurement endpoint so provides a useful comparison of chemical potency^57^. Once the NEC is exceeded, the pattern of mortality over time depends on the toxicokinetics (i.e the elimination rate *k*_e_) and toxicodynamics (i.e. the killing rate *k*_k_) of the chemical. For slowly accumulating chemicals, the full hazard may not be realised even following life-time exposure if the life-span of the organism is not sufficient to reach internal equilibrium. Time to reach a certain fraction (x) saturation of the internal equilibrium concentrations can also be derived from the elimination rate as: t_x_ = −(1/ke) ln(1 − x), with 95% saturation chosen as the value for comparison. The killing rate provides a measure of damage due to the accumulated chemical. If this rate is low, then also the full effect on survival may not be realised within the organism life-time and so the full theoretical extent of mortality may not be achieved.

DEBtox parameters were calculated from the time-course of survival and used to estimate LC_5_ and LC_50_ values for time periods relevant to laboratory exposure (e.g. 48 h, 96 h, 240 h) and also for prediction of extended periods of 720 h and 2160 h corresponding to 30 d and 90 d; these values being equivalent to the approximated life-span of an individual adult worker bee during the peak foraging season and when overwintering, respectively although not accounting for between seasons sensitivity^58^.

## Additional Information

**How to cite this article**: Hesketh, H. *et al*. Extending standard testing period in honeybees to predict lifespan impacts of pesticides and heavy metals using dynamic energy budget modelling. *Sci. Rep.* **6**, 37655; doi: 10.1038/srep37655 (2016).

**Publisher's note:** Springer Nature remains neutral with regard to jurisdictional claims in published maps and institutional affiliations.

## Supplementary Material

Supplementary Information

## Figures and Tables

**Table 1:** Rationale for chemical selection based on mode of action and current concerns about agrochemicals and trace pollutants in the environment.

Chemical	Primary use	Class	Mode of action	Background for selection
Dimethoate	Spray and topical insecticide	Organophosphate	Binds to and irreversibly inactivates acetylcholinesterase. Active ingredient is a serine protease that hydrolyses the neurotransmitter acetylcholine at the synaptic junction.	Used to control a range of pests and the OEC reference toxicant used in routine testing honeybees and other arthropod species)^14^. Potential for increased OP use as other pesticides are withdrawn from use
2,4-Dichlorophenoxyacetic acid	Herbicide	Synthetic auxin	Mimics plant growth hormone auxin	A synthetic auxin herbicide, widely used for control of broadleaf weeds
Clothianidin	Systemic insecticide	Chloro-nicotinyl	Binds to nicotinic acetylcholine receptors to trigger activation and nervous overstimulation	A neonicotinoid insecticide which is used as a systemic insecticide and seed dressing against a wide variety of agricultural pests
Tau-fluvalinate	Varroacide and pesticide on oilseed rape	Pyrethroid	Binds to voltage-gated sodium channels in order to depolarise nerves. Relatively low binding to receptor in bees	A synthetic pyrethroid insecticide used against agricultural pests and extensively for *Varroa destructor* mite control in bee hives (high probability of coexposure). This insecticide is considered relatively non-toxic to bees, but is reported as being highly persistent in bee hives^59^ and has shown evidence of synergism when considering P450-mediated detoxification pathways^46^
Propiconazole	Fungicide	Conazole	Sterol biosynthesis inhibition by blocking the cytochrome P450 14-alpha-demethylase	A sterol inhibiting and commonly used fungicide in rape from a class identified as a potential synergist when part of a co-exposure A sterol inhibiting broad spectrum fungicide from a class of fungicides that have been reported as a potential synergists with other chemicals (Cedergreen 2006)^60^
Cadmium	Environmental contaminant	Heavy metal	Induces genomic instability through complex and multifactorial mechanisms.	A non-essential heavy metal that is a known widespread toxic environmental contaminant with long-term and diverse toxic effects.
Arsenic	Historic use as pesticide	Metalloid	Co-factor substitution in metalloproteins, oxidative stress effect on the structure and functions of plasma membranes and effects on macromolecules including genotoxicity.	Widespread non-essential metal contaminant in soils, water and dust especially in agricultural areas due to past pesticide use and its presence in trace amounts in phosphate fertiliser. Known to be highly toxic and affect the genome. Included in study to provide cross validation for other ecotoxicity tests.

**Table 2:** Toxicity of five chemicals to *Apis mellifera* spp.: Probit estimates of oral LC_50_ values with 95% confidence limits in parentheses.

	Dimethoate	Clothianidin	Propiconazole	Cadmium	Arsenic
Probit calculated 48 h LC_50_ ± 95% CI mg/L	2.42 (1.96–2.89)	0.158 (0.089–0.227)	nc	18.355 (9.082–27.629)	25.675 (22.222–29.129)
Probit calculated 96 h LC_50_ ± 95% CI mg/L	1.16 (0.95–1.38)	0.079 (0.059–0.010)	nc	3.697 (0–11.916)	13.558 (11.999–15.116)
Probit calculated 240 h LC_50_ ± 95% CI mg/L	0.62 (0.46–0.77)	0.028 (0.018–0.038)	nc	nc	4.030 (3.314–4.745)
Probit calculated LC_50_ 48 h: 96 h	2.08	2.00	nc	4.99	1.88
Probit calculated LC_50_ 48 h: 240 h	3.90	*5.64*	nc	nc	*6.35*

DEB Calculated 48 h LC_50_ mg/L	1.55	0.0257	1363	37.68	22.04
DEB Calculated 96 h LC_50_ mg/L	0.83	0.0243	738	13.80	10.36
DEB Calculated 240 h LC_50_ mg/L	0.54	0.0240	403	4.52	5.65
DEB Calculated 720 h LC_50_ mg/L	0.45	0.0240	299	1.39	4.48
DEB Calculated 2160 h LC_50_ mg/L	0.42	0.0240	292	0.45	4.27
DEB LD_50_ 48 h: 240 h	2.87	1.07	3.38	*8.34*	3.90
DEB LD_50_ 48 h: 720 h	3.44	1.07	4.56	**27.11**	4.92
DEB LD_50_ 48 h: 2160 h	3.69	1.07	4.67	**83.73**	*5.16*

DEBtox parameter estimates for 48 h, 96 h and 240 h LC_50_ values are presented as estimate effects for a typical short-term laboratory bioassay (48 h, 96 h), extended duration bioassay (240 h), summer worker bee life-time (720 h) and winter bee life-time (2160h). The relative change of toxicity is estimated at the comparison of 48 h: 96 h, 48 h: 240 h for Probit and at 48 h: 240 h, 48 h: 720 h and 48 h: 2160 h for DEBtox estimated values. Values could not be calculated for tau-fluvalinate or 2,4-D as mortality levels were insufficient to establish any dose-response relationship. LC_50_ values varying between time-points by a factor of >5 but <20 are shown in italic font and LC_50_ values varying between time-points by a factor >20 are shown in bold font.

**Table 3:** 

	Blank killing rate (h^−1^)	No Effect Concentration (mg/L)	Elimination rate (h^−1^)	Killing rate (mg/h)
Dimethoate	0.001	0.41	0.04	0.03
Clothianidin	0.0018	0.024	0.073	0.124
Tau-fluvalinate	0.0009	—	—	—
2,4-D	0.0005	—	—	—
Propiconazole	0.001	292	0.006	0.0036
Cadmium	0.003	0	0.037	0.00072
Arsenic	0.00075	4.2	0.015	0.0053

DEBtox parameter values for model fits for the effects of seven chemicals on survival over time for Apis mellifera spp. blank killing rate is measure of the rate of background mortality in a population not subject to exposure; the No Effect Concentration (NEC) is a time-independent toxicological threshold below which no effects occur even over infinite exposure time; the elimination rate is a rate parameter determining when the equilibrium between internal and external concentration is reached in time; the killing rate is the toxic potency of the compound (once the NEC is exceeded) expressed in relation to the environmental concentration and time.
