# Supplementary material for: Extending standard testing period in honeybees to predict lifespan impacts of pesticides and heavy metals using dynamic energy budget modelling
Source: Sci Rep. 2016 Dec 20;6:37655. doi: 10.1038/srep37655 (PMC5171639; doi:10.1038/srep37655)
Supplement: Supplementary Information [file srep37655-s1.doc]

Supplementary Table S1: GLM analysis.

Explanatory variables for all analyses were transformed log_10_ chemical concentration+1 and colony, with proportion mortality with colony as response variable. A full model with all interaction terms was fitted and then simplified by sequentially removing terms with high, non-significant, *p-*values.

**Dimethoate**

48h Log concentration F_1, 18_=16.15, p=0.001 Colony F_3,18_=0.81, p=0.506

96h Log concentration F_1,18_=28.99, p<0.001 Colony F_3,18_=0.92, p=0.451

240h Log concentration F_1,18_=15.45, p=0.001 Colony F_3,18_=0.07, p=0.974

**Clothianidin**

48h Log concentration F_1, 20_=5.43, p=0.032 Colony F_2,20_=4.41, p=0.029

96h Log concentration F_1,20_=16.26, p=0.001 Colony F_2,20_=4.31, p=0.031

240h Log concentration F_1,20_=27.59, p<0.001 Colony F_2,20_=1.59, p=0.232

**Propiconizole**

48h Log concentration F_1, 18_=0.49, p=0.494 Colony F_3,18_=0.64, p=0.600

96h Log concentration F_1,19_=2.85, p=0.108 Colony F_3,19_=1.31, p=0.300

240h Log concentration F_1,19_=8.62, p=0.008 Colony F_3,19_=0.60, p=0.624

**Arsenic**

48h Log concentration F_1, 23_=100.02, p<0.001 Colony F_3,23_=0.36, p=0.786

96h Log concentration F_1,23_=128.74, p<0.001 Colony F_3,23_=0.28, p=0.839

240h Log concentration F_1,22_=15.26, p=0.001 Colony F_3,22_=1.09, p=0.375

**Cadmium**

48h Log concentration F_1, 22_=24.63, p<0.001 Colony F_3,22_=3.56, p=0.031

96h Log concentration F_1,22_=28.65, p<0.001 Colony F_3,22_=2.53, p=0.083

240h Log concentration F_1,21_=12.13, p=0.002 Colony F_3,21_=1.44, p=0.258

**2,4-D**

48h Log concentration F_1, 19_=0.19, p=0.669 Colony F_3,19_=0.96, p=0.432

96h Log concentration F_1,19_=0.21, p=0.651 Colony F_3,19_=0.39, p=0.791

240h Log concentration F_1,19_=0.48, p=0.497 Colony F_3,19_=0.48, p=0.698

**Taufluvalinate**

48h Log concentration F_1,19_ = 1.75, p = 0.201 Colony F_3,19_ = 0.86, p = 0.476

96h Log concentration F_1,19_ = 0.01, p = 0.916 Colony F_3,19_ = 1.64, p = 0.214

240h Log concentration F_1,19_ = 0.66, p = 0.427 Colony F_3,19_ = 2.53, p = 0.088

Supplementary Table S2. Probit estimated LD_50_ values (48 h, 96 h, 240 h) based on the time-course effects of four chemicals (dimethoate, clothianidin, cadmium, arsenic) on survival over time for *Apis mellifera* (note: values for tau-fluvalinate, propiconazole, 2,4-D not calculated as there was no dose related effect for these chemicals) based on total consumed dose (mg/bee) for each time point and total consumed dose per mg bee body weight.
